# Effects of Tributyrin Supplementation on Growth Performance, Insulin, Blood Metabolites and Gut Microbiota in Weaned Piglets

**DOI:** 10.3390/ani10040726

**Published:** 2020-04-22

**Authors:** Stefania Sotira, Matteo Dell’Anno, Valentina Caprarulo, Monika Hejna, Federica Pirrone, Maria Luisa Callegari, Telma Vieira Tucci, Luciana Rossi

**Affiliations:** 1 Department of Health, Animal Science and Food Safety, Università degli Studi di Milano, via Trentacoste 2, 20134 Milan, Italy; stefania.sotira@unimi.it (S.S.); valentina.caprarulo@unimi.it (V.C.); monika.hejna@unimi.it (M.H.); luciana.rossi@unimi.it (L.R.); 2 Department of Veterinary Medicine, Università degli Studi di Milano, Via dell’Università 6, 26900 Lodi, Italy; federica.pirrone@unimi.it; 3 Department of Sustainable Food Process, Faculty of Agriculture, Food and Environmental Sciences, Università Cattolica del Sacro Cuore, via Emilia Parmense 84, 29122 Piacenza, Italy; marialuisa.callegari@unicatt.it; 4 Freelance Veterinarian, Via Fratelli Bandiera 28, 46100 Mantova, Italy; telmatucci@gmail.com

**Keywords:** pig nutrition, antibiotic alternatives, feed additives, butyrate, lactobacilli

## Abstract

**Simple Summary:**

In animal farming, alternatives to antibiotics are required due to the increase of antimicrobial resistance. In this contest, tributyrin showed the ability to promote gut health, to modulate gut microbiota and to improve protein digestibility, leading also to higher growth performance. However, although the mode of action of tributyrin on the intestinal epithelial cells has been partially explained, its effects on lipid and protein metabolism needs to be investigated. This paper provides information about the influence of tributyrin on production traits, blood parameters, faecal microbiota and faecal protein excretion in weaned piglets.

**Abstract:**

The aim of this study was to investigate the effects of tributyrin supplementation on the production traits, the main metabolic parameters and gut microbiota in weaned piglets. One hundred and twenty crossbred piglets (Large White × Landrace) were randomly divided into two experimental groups (six pens each; 10 piglets per pen): the control group (CTRL), that received a basal diet, and the tributyrin group (TRIB) that received the basal diet supplemented with 0.2% tributyrin. The experimental period lasted 40 days. Production traits were measured at days 14, 28 and 40. A subset composed of 48 animals (*n* = 4 for each pen; *n* = 24 per group) was considered for the evaluation of serum metabolic parameters and hair cortisol by enzyme-linked immunosorbent assay (ELISA), and faecal microbiota by real-time polymerase chain reaction (PCR). Our results showed that the treatment significantly increased body weight (BW) at day 28 and day 40 (*p* = 0.0279 and *p* = 0.0006, respectively) and average daily gain (ADG) from day 28 to day 40 (*p* = 0.046). Gain to feed ratio (G:F) was significantly higher throughout the experimental period (*p* = 0.049). Even if the serum parameters were in the physiological range, albumin, albumin/globulin (A/G) ratio, glucose and high-density lipoproteins (HDL) fraction were significantly higher in the TRIB group. On the contrary, tributyrin significantly decreased the urea blood concentration (*p* = 0.0026), which was correlated with lean gain and feed efficiency. Moreover, serum insulin concentration, which has a regulatory effect on protein and lipid metabolism, was significantly higher in the TRIB group (*p* = 0.0187). In conclusion, this study demonstrated that tributyrin can be considered as a valid feed additive for weaned piglets.

## 1. Introduction

Even if antibiotics remain an essential tool for treating animal diseases, innovative feed additives are required in order to reduce their use in livestock due to increased antimicrobial resistance. In pig farming, the weaning phase is characterized by high levels of stress resulting in decreased feed intake, growth retardation and a higher tendency to develop gastrointestinal diseases [1,2] that may require the use of antimicrobial compounds. Thus, alternatives include the use of feed supplements able to improve general health status by modulating the digestive process and the intestinal microbiota, such as probiotics, prebiotics and organic acids [3,4,5]. In light of this, short chain fatty acids (SCFA) play a fundamental role in modulating the intestinal microbial population and in promoting the digestion phase [6]. Butyrate is a SCFA that is produced by bacteria in the gut [7]. Aside from its primary function as an energy source for colonocytes, it is a strong mitosis promoter and a differentiation agent for intestinal epithelial cells [8], as it acts as a histone deacetylase (HDAC) inhibitor [9]. It showed also in vitro positive effects on hepatocytes [10]. Moreover, it has a strong antibacterial activity against both Gram-negative and Gram-positive pathogens [11] and therefore proves to be a valid aid for gut health maintenance. In pigs, different studies showed the improvement of growth performance, the repair of damaged intestinal tissues and the improvement of protein digestibility [12,13,14]. However, a decreased feed intake associated with its strong odour has been observed when high levels of butyrate were included in the diet. [15]. Tributyrin is a valid alternative to butyrate, as one molecule of tributyrin releases three molecules of butyrate directly in the small intestine, thus butyrate is rapidly adsorbed. Supplementation of tributyrin showed different in vivo positive effects on growth performance and gut health, also after lipopolysaccharide challenge [16,17,18]. The higher growth performance and the improvement of protein digestibility suggest that tributyrin could modulate protein and lipid metabolism. Thus, blood metabolites, insulin and leptin, which are positively correlated with body weight and with adipose and also muscle mass, could be modulated by tributyrin supplementation. However, no other studies investigated the effects of in-feed tributyrin on blood metabolites (such as glucose, urea and HDL), insulin, leptin and cortisol in healthy piglets. Thus, the aim of this study was to determine the effects of tributyrin supplementation on the production traits and nutrient metabolism in piglets reared in a conventional herd. In particular, the main blood metabolites, insulin, leptin, hair cortisol and protein content in faecal samples were analysed. Moreover, the effects of tributyrin on gut microbiota were evaluated.

## 2. Materials and Methods 

### 2.1. The In Vivo Trial 

The in vivo trial, complied with Italian regulation on animal experimentation and ethics (DL 26/2014) in accordance with European regulation (Dir. 2010/63), was approved by the animal welfare body of University of Milan (authorization number 31/2019) and performed in an intensive conventional herd, located in Lombardy (Italy), free from diseases according to the ex A-list of the World Organization for Animal Health (Aujeszky’s disease, atrophic rhinitis, transmissible gastroenteritis, porcine reproductive and respiratory syndrome and salmonellosis). 

One hundred and twenty crossbred piglets (Large White × Landrace) weaned at 28 ± 2 days were randomly allotted into two experimental groups, with similar conditions to those under which commercial farm animals were kept before the first day of the trial (six pens per group; 10 piglets each, 50% female and 50% male) After one week of adaptation, the control group (CTRL) received a basal diet, while the tributyrin group (TRIB) received the same basal diet supplemented with 0.2% of tributyrin (Ferraroni Mangimi SpA, Bonemerse, Italy). The iso-energetic and iso-proteic diet provided fulfilled the National Research Council NRC [19] requirements (Table 1). The experimental period lasted 40 days, considering day 0 the first day the two groups received the two different experimental diets. 

Piglets of both experimental groups were reared in a unique room, with environmental controlled conditions (temperature: 27 °C; humidity: 60%). In particular, the room had an unobstructed floor area available to each weaner piglet of 0.40 m^2^, according to the Directive 2008/120/EC. Feed and water were provided ad libitum.

### 2.2. Zootechnical Evaluation

The body weight was individually measured on day 0, day 14, day 28 and day 40. Average daily feed intake (ADFI) was calculated weekly by weighting the feed refuse of each pen (experimental unit for the zootechnical analyses). Average daily gain (ADG) was calculated from day 0 to 14, from day 14 to 28 and from day 28 to 40. Gain to feed ratio (G:F) was calculated from day 0 to 14, from day 14 to 28 and from day 28 to 40. The health status of the piglets was monitored daily and mortality was registered.

### 2.3. Protein Content in Faecal Samples

The Crude Protein (CP) in faecal samples, strictly depending on dietary protein level, was analysed in order to evaluate the protein excretion as indirect parameter to estimate the protein utilization and digestibility. Faecal samples were individually collected from rectal ampulla from a subset of animals (*n* = 48, 4 piglets for each pen, 50% female and 50% male) on day 40 and stored at −20 °C for further analyses. The samples were analysed following Official methods of analysis [20]. In particular, dry matter (DM) was obtained by inserting 2 g of faecal samples in previously weighed aluminium bags and dried in a forced-air oven at 105 °C for 24 h. The dried samples were then weighted and analysed for the protein content with the Kjeldahl method [20]. 

### 2.4. Blood Sample Collection and Biochemical Analyses

Blood was collected from the jugular vein from a subset of animals (*n* = 48, 4 piglets for each pen, 50% female and 50% male) randomly selected in each treatment group on day 40. Blood samples were collected into vacuum tubes from each animal and maintained for 2 h at room temperature. All samples were centrifuged at 3000 rpm for 10 min at 4 °C. Serum was removed and the aliquots were stored at −20 °C for further analysis. The concentration of total protein (g/L), albumin (g/L), globulin (g/L), albumin/globulin (A/G ratio), urea (mmol/L), alanine aminotransferase (ALT-GPT, IU/L), aspartate aminotransferase (AST-GOT) IU/L, phosphatase alkaline (ALP) UI/L, total bilirubin (μmol/l), glucose (mmol/L), total cholesterol mmol/L, high-density lipoproteins (HDL) and low-density lipoproteins (LDL) fraction, calcium mmol/L, phosphorus (mmol/L), magnesium (mmol/L) were determined by multiparametric auto-analyser for clinical chemistry (ILab 650; Instrumentation Laboratory Company, Lexington, MA, USA). 

### 2.5. Insulin and Leptin Evaluation by Enzyme-Linked Immunosorbent Assay (ELISA)

Blood was collected from the jugular vein of the piglets after one hour of fasting, on day 40 during the morning and within one hour in order to have homogeneous conditions and the most representative parameters. Serum insulin and leptin were evaluated through enzyme-linked immunosorbent assay (ELISA) kits specific for pigs (CEA44Po and SEA084Po, Cloud-Clone corp, Katy, TA, USA) according to manufacturer instructions. Samples (*n* = 24, 2 piglets per pen, 50% female and 50% male) were diluted (1:5) for leptin determination, as suggested by the instructions, and tested as fresh weight for insulin. Absorbances were measured with a microplate reader at 450 nm (Bio-Rad 680 microplate reader; Bio-Rad Laboratories, Inc., Hercules, CA, USA) and concentrations were calculated according to the respective standard curve. 

### 2.6. Hair Cortisol Extraction and Assay

Hair samples were collected in one sampling time on the back, in the rump region, from a subset of animals (*n* = 48, 4 piglets per pen, 50% female and 50% male) randomly selected on day 40. According to Casal et al. [21] the hair follicle was not included in the sample, to avoid contamination from blood, and for the potential endocrine activity. The four samples were pooled in one sample that was then analyzed, for a total of 12 samples. Cortisol extraction was performed following the method of Burnett et al. [22], and partially modified according to Koren et al. [23]. At the end of extraction, the samples were centrifuged in a microcentrifuge (10 min; 3000 rpm) and 0.8 mL of the supernatants were dried using a nitrogen flow at a temperature of 45 °C and stored at −20 °C until the time of analysis. 

Hair cortisol concentration was assessed using an Expanded Range High Sensitivity Salivary Cortisol ELISA kit (Salimetrics, State College, PA, USA) following previously validated protocols [21]. Concentrations were calculated using a Labisystem Multiskan Ex (Midland, ON, Canada) microplate reader according to the relevant standard curves. Intra- and inter coefficient of variances were 8.8% and 9.3%, respectively. 

### 2.7. DNA Extraction and Real-Time Polymerase Chain Reaction (PCR) to Determine Gut Microbiota 

Bacterial DNA was extracted as previously reported by Patrone et al. [24]. Copy numbers of the 16S rRNA gene from *Escherichia coli, Enterobacteriaceae, Bifidobacterium* spp. and *Lactobacillus* spp. were quantified using previously reported primers [25,26,27]. Quantification was carried out in triplicate (n = 24, 2 piglets per pen, 50% female and 50% male) using the LightCycler 480 Instrument II (Roche Diagnostics, Monza, Italy). *Bifidobacterium* spp., *Lactobacillus* spp. and *Enterobacteriaceae* were quantified using the KAPA SYBR^R^ FAST (Kapa Biosystems, Inc; Wilmington, MA, USA) containing a 300 nM final primer concentration. Instead, *E. coli* was quantified using the KAPA Probe FAST Master mix (Kapa Biosystems, Inc; Wilmington, MA, USA) containing 500 nM of primers and 100 nM of probe (final concentration). Primers and probe used for the quantification of *E. coli* were described by Penders et al. [25]. *Bifidobacterium infantis* ATCC 15697D and *E. coli* ATCC 700926D-5 genomic DNAs, used for preparing standard curves, were provided by the American Type Culture Collection (ATCC). Genomic DNA of *Lactobacillus fermentum* DSM20052 was obtained by extracting 5 mL of activated culture using the Genomic DNA extraction Kit (Promega) and quantified with a Qubit™ fluorometer (Invitrogen, Milan, Italy). Standard curves were obtained by 10-fold dilutions of genomic DNA for each reference genomic DNA. Results were obtained as ng of target/ng of total bacterial DNA and logarithmic transformation of real-time polymerase chain reaction (PCR) data was performed to achieve normal distribution. 

### 2.8. Statistical Analysis 

Data were analyzed using generalized linear mixed model through Proc GLIMMIX of SAS 9.4 (SAS Inst. Inc., Cary, NC, USA), and the repeated measures over time were included in the RANDOM statement. The model included the fixed effect of treatments (TRT), experimental day (D) and the interaction between the two factors was evaluated (TRT × D). The model also included the random effects of piglets nested within treatments. Tukey–Kramer studentized adjustments were used to separate treatment means within the two-way interactions. Within significant two-way interactions, slice option was used to separate means within a specific treatment and experimental days. Results are presented as least square means (LSMEANS) ± standard error (SE). Student *t*-test was used to analyse data obtained from the ELISA assays and real-time PCR. Results are presented as means ± standard error (SE). Means were considered different when *p* ≤ 0.05. 

## 3. Results

### 3.1. Zootechnical Evaluation

No significant differences in the ADFI during the entire experimental period (*p* = 0.0946) were observed. However, the treatment had a positive effect on the BW, with significantly higher weights at day 28 and day 40 (*p* = 0.0279 and *p* = 0.0006, respectively). The ADG was significantly higher from day 28 to day 40 in TRIB group compared to the CTRL group (*p* = 0.046). Moreover, during the whole experimental period a significantly higher G:F in the TRIB group was noticed (*p* = 0.049) (Table 2). Only one piglet of the CTRL group died at day 7. By contrast, no mortality was observed in the TRIB group. No differences were observed between male and female in the parameters analyzed. 

### 3.2. Protein Content in Faecal Samples

The average protein content in faecal samples was significantly lower in the TRIB group compared to the CTRL group (19.5 ± 1.91 and 22.8 ± 1.48% of DM, respectively; *p* = 0.039, DF = 3). The data are expressed as mean ± SD. 

### 3.3. Blood Sample Collection and Biochemical Analyses

The serum concentrations of total protein, globulin, AST-GOT, ALT-GPT, phosphatase alkaline, total bilirubin, total cholesterol, calcium, phosphorus, magnesium and the LDL fraction did not significantly differ between the two experimental groups (Table 3). Dietary addition of tributyrin significantly increased the serum concentration of albumin (*p* = 0.0002), A/G (*p* = 0.0117), glucose (*p* = 0.0396) and HDL fraction (*p* = 0.0349). Moreover, the animals fed the diet supplemented with 0.2% tributyrin showed a significant decrease in the urea blood concentration (*p* = 0.0026). 

### 3.4. Insulin and Leptin Evaluation

**Figure 1:**
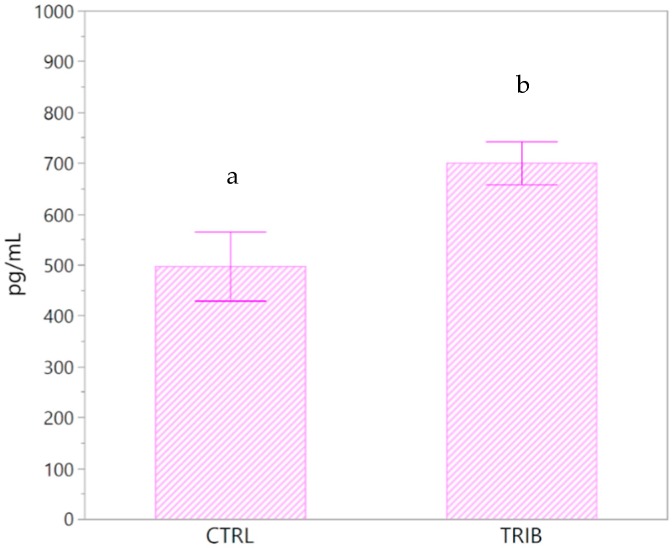
Serum insulin concentrations (pg/mL) in the control group (CTRL) and in the group of piglets fed a diet supplemented with 0.2% tributyrin (TRIB). Data are presented as the mean ± SE. a,b: significant differences between groups (*p* = 0.0187).

Serum leptin did not differ between TRIB and CTRL groups (1215.6 ± 112.29 pg/mL and 1228.6 ± 121.09 pg/mL, respectively. *p* = 0.6649, DF = 25.8). On the contrary, insulin levels were significantly different between the treatment and the control groups (*p* = 0.0187, DF = 21.8). The average level of insulin in the TRIB group was 700.5 ± 42.35 pg/mL, while the CTRL group was 497.2 ± 67.84 pg/mL (Figure 1).

### 3.5. Cortisol Concentration

**Figure 2:**
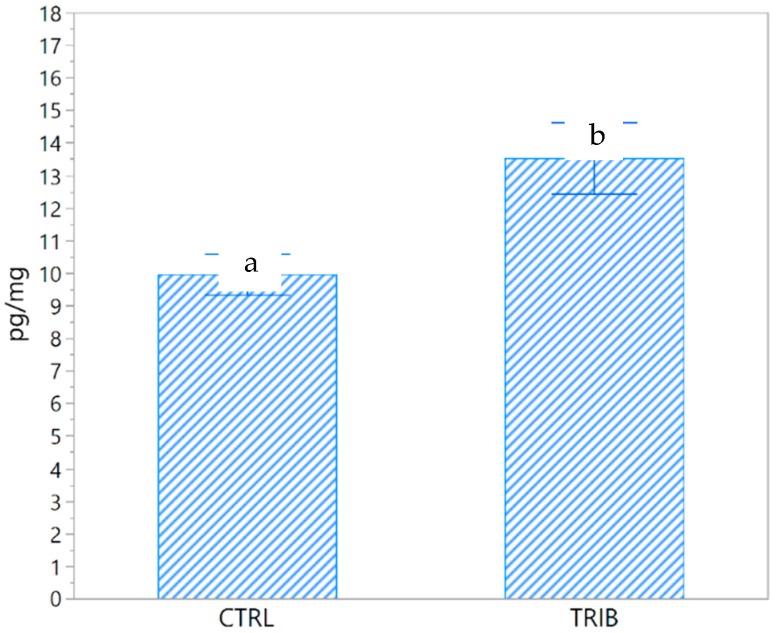
Hair cortisol concentrations (pg/mg) in the control group (CTRL) and in the group of piglets fed a diet supplemented with 0.2% tributyrin (TRIB). Data are presented as the mean ± SE. a,b: significant differences between groups (*p* = 0.018).

Hair cortisol differed significantly between the TRIB and the CTRL groups (*p* = 0.018, DF = 10). The average level of cortisol with the standard error (SE) of the CTRL group was 9.9 ± 0.63 pg/mg, while the TRIB group had an average concentration of 13.5 ± 2.22 pg/mg (Figure 2). 

### 3.6. DNA Extraction and Real-Time PCR to Determine Gut Microbiota 

**Figure 3:**
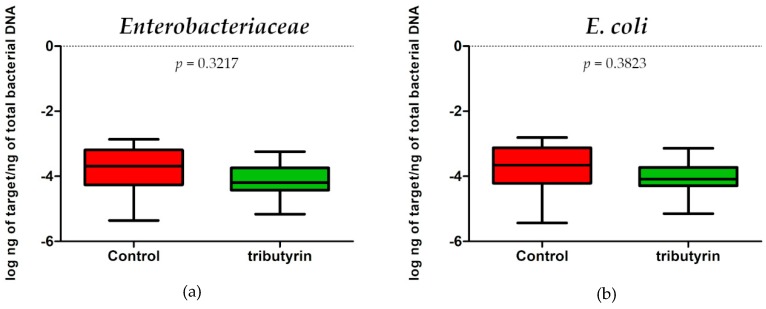
(**a**) *Enterobacteriaceae* abundance expressed as log ng of target/ng of total bacterial DNA in the faecal samples of the control group and in the group of piglets fed a diet supplemented with 0.2% tributyrin. (**b**) *E. coli* abundance expressed as log ng of target/ng of total bacterial DNA in the faecal samples of the control group and in the group of piglets fed a diet supplemented with 0.2% tributyrin.

**Figure 4:**
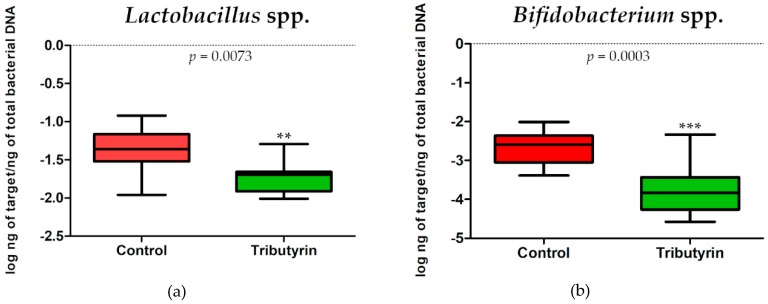
(**a**) *Lactobacillus* spp. abundance expressed as log ng of target/ng of total bacterial DNA in the faecal samples of the control group and in the group of piglets fed a diet supplemented with 0.2% tributyrin. (**b**) *Bifidobacterium* spp. abundance expressed as log ng of target/ng of total bacterial DNA in the faecal samples of the control group and in the group of piglets fed a diet supplemented with 0.2% tributyrin.

No significant differences in *E. coli* (*p* = 0.3823; DF = 11) and *Enterobacteriaceae* (*p* = 0.3217; DF = 11) content were detected between faecal samples of the two groups of animals (Figure 3). Instead, a significant reduction of lactobacilli (*p* = 0.0073; DF = 11) and bifidobacteria (*p* = 0.0003; DF = 11) was found in the TRIB group (Figure 4). 

## 4. Discussion

The present study demonstrated that the tributyrin supplementation can influence positively the growth performance of healthy weaned piglets, showing the feeding effect on body weight, average daily gain, and G:F ratio. Results obtained in our study were consistent with the finding of Hou et al. [28] who found that 0.5% tributyrin in diet improved the growth rate and feed conversion ratio of piglets. Murray [29] showed that the inclusion of tributyrin at the inclusion rate of 0.25% and 0.5% increased the growth and muscle hypertrophy related to its HDAC inhibition activity. Considering our results, we can suppose that in addition to the trophic effect on muscles, the higher weights were also induced by the trophic effect of tributyrin on the gastrointestinal tract, thus resulting in a larger absorptive surface and better growth performance. The positive effects of the dietary tributyrin are related to the release of three molecules of butyrate directly in the intestine, which is a strong mitosis promoter [8] and provides a positive effect on intestinal cell proliferation, increasing crypt depth, villi length and mucosa thickness in jejunum and ileum [30]. The increased gut health enhances also the absorption capacity, which is of greater biological value during the weaning period [14,30]. In fact, post weaning is a critical phase in pig livestock as several stressors can compromise the health status and the productive parameters of the piglets, causing limitations in the digestive and absorptive capacity [31]. The supplementation of organic acids reduces also the gastric pH, resulting in the increase of pepsin activity, gastric retention time and improved protein digestion [32]. However, the use of butyrate instead of tributyrin led to the rapid absorption of butyrate at stomach level, which causes a reduced availability in the intestine, where it plays a pivotal role [8]. Studies in piglets showed a positive effect of dietary butyrate on growth at 0.8% inclusion levels [33], even if Lallès et al. [34] showed a decreased feed intake in piglets following increased inclusion levels of butyrate, which may be associated with its pungent odour. In this study, the dietary inclusion of 0.2% tributyrin increased the growth performance without altering the feed intake, confirming both its good tolerability and its ability of influence the feed efficiency. 

Nutrition is a very important aspect of pig production which could affect blood metabolites [35]. In our study, the increase of serum glucose observed in the TRIB group compared with the CTRL group is probably associated with the higher body weights and growth performance. In fact, the glucose level is within the physiological range, according to other findings [36]. Moreover, we can suppose that butyrate, which possesses a note histone deacetylase inhibition activity [37], promoted β-cells development, proliferation and function as well as improved glucose homeostasis [38,39]. For these reasons, it has a key role in maintaining adequate glucose level in blood [40].

The observed increase of HDL fraction occurred without affecting the total cholesterol content in blood. HDL exhibits a variety of anti-atherogenic effects, including anti-inflammatory and antioxidant activity, and the promotion of cholesterol efflux, which not only affects foam cell formation but also positively affects the reverse cholesterol transport [41,42]. Nazih et al. [43] demonstrated that butyrate was the only fatty acid that significantly increased the synthesis and the secretion of ApoA-IV protein, which is a major component of HDL and promotes the synthesis of ApoA-IV-containing HDL [44]. Xiong et al. [45] showed that tributyrin supplementation in LPS-challenged broilers increased serum HDL compared to LPS-challenged broilers without any feed supplement. Our study, together with previous results, showed that tributyrin might lead to positive effects on blood lipid regulation. ALT-GPT and AST-GOT were considered as indicator of potential liver damage. Considering our data, the tributyrin supplementation had no negative effects on hepatic functionality.

Albumin and as a consequence the albumin to globulin ratio was significantly higher in the TRIB group compared to the CTRL group. According to Dvoràk [46], albumin and A/G quotient increase gradually from birth until the post-weaning period. In particular, the better-growing piglets mostly had higher albuminemia, suggesting that nutrition, body growth and albumin synthesis are interdependent in piglets. In fact, albumin has been always linked to the nutritional status, as it rapidly increases after feeding and has always low levels during malnutrition [47]. In pigs, albumin is considered one of the most important predictors of performance, especially average daily gain and feed conversion ratio [48]. In accordance with these finding and Elbers et al. [49], our study demonstrated that piglets with higher ADFI and ADG had higher albumin levels. Moreover, in our study, insulin was significantly higher in the TRIB group compared to the CTRL group. Albumin is strongly related to insulin synthesis [50,51] and it is well known that insulin is associated with muscle protein synthesis [52,53]. Moreover, the data in our study were in the physiological range [49]. The same author demonstrated that piglets with higher serum albumin had also higher daily weight gain, in line with our study. 

Urea is a waste product physiologically produced by the liver when the body breaks down proteins and high levels in blood are related to kidney or liver problems. Our results revealed that tributyrin significantly decreased the serum urea in piglets. Usually, low urea nitrogen is associated with protein deficiency in the diet [54] however the formulation of our diet fit completely the nutrient requirement of piglets. In the last decade, different studies demonstrated that plasma urea nitrogen (PUN) concentration has also a strong and inverse relationship with the lean tissue growth suggesting that the better-growing piglets have the lowest PUN values. Whang and Easter [55] also demonstrated that blood urea nitrogen (BUN) in pigs is negatively correlated with lean gain and feed efficiency. Urea production should reflect not only alterations in the dietary intake of proteins but also an animals’ ability to retain dietary nitrogen in the body indicating the effective protein utilization [56]. This data, in line with the increased growth performance, is also reflected in the protein content of dried faeces and, together, these data could suggest better protein absorption and utilization. It is well known that the piglets have a relatively high gastric pH due to the scarce secretion of hydrochloric acid and pancreatic enzymes [57]. This typical condition led to limitations in the absorption and digestion processes and strategies to decrease the pH are used to overcome these problems. Organic acids can decrease the gastric pH [58] particularly tributyrin, resulting in the conversion of the pepsinogen into pepsin, thus increasing the activity of the proteolytic enzymes and improving protein digestion [16] Moreover, an in vitro experiment showed that butyrate enhanced the expression and activity of the peptide transporter PEPT1, located on the enterocytes, that has a key role in the protein-nitrogen absorption [59]. We suppose that in our study tributyrin ameliorated the protein absorption and utilization by the summation of different mechanisms: it principally lowered the gastric pH, thus activating the proteolytic enzymes; it increased the absorptive surface, due to its trophic effect on the gastrointestinal tract and it probably enhanced the activity of peptide transporter on the enterocytes surface. 

Insulin increases rapidly after feeding and plays a key role in regulating the assimilation of nutrients. Studies in pigs in developmental ages showed that the increase in stimulation of muscle protein synthesis in piglets is mediated by the rise of insulin in a dose-dependent way and that this response declines with adult age [52,53]. According to He et al. [60], tributyrin exerted also a regulatory effect also on lipid metabolism, as demonstrated also by our findings. In line with these literature results, our study showed that tributyrin increased the levels of insulin and this result is probably related to the higher growth performance. Moreover, several studies showed that SCFAs stimulate insulin secretion in ruminants [61] In monogastrics, butyrate increases insulin sensitivity, decreasing insulin resistance [60]. SCFAs affect pancreatic beta-cell function by directly acting as HDAC inhibitors (promoting β-cell development, proliferation, and differentiation) or indirectly, leading to insulin release [62]. However, the effects of SCFAs on insulin secretion are controversial, as some authors report that there might be no direct effect at all [63]. 

The increase of the cortisol concentration in stressful situations is an adaptation response of the organism to a changing environment. Studies in sheep demonstrated that ruminal infusion of a mixture of SCFAs (acetate, propionate and butyrate) increased plasma cortisol [64]. In piglets, Weber and Kerr [65] found that LPS-challenged piglets fed butyrate 0.2% had higher serum cortisol if compared to LPS-challenged piglets fed a basal diet. However, it is unknown whether butyrate infusion alone would elevate cortisol levels. Hillmann et al. [66] showed that mean cortisol levels in growing pigs were affected by the weight, and daily peaks of cortisol were higher with increasing weights. It is probable that the results of our study were related to higher body weights and not with tributyrin supplementation, however further studies are needed to better understand the role of tributyrin on cortisol level. 

It is well known that dietary, environmental and social stresses induced by weaning transition are associated with microbial shifts in piglets [67]. Therefore, post-weaning is a critical period as it is associated with diarrhea and depression of growth performance. Gut microbiota plays a key role in both animal growth performance and healthy status. In this study, only *E. coli*, *Enterobacteriaceae*, *Lactobacillus* spp. and *Bifidobacterium* spp. were quantified by real-time PCR. We selected these bacterial groups because pathogens are present among the *Enterobacteriaceae* and, in particular, strains of *E. coli* that are involved in diarrhea and other diseases [68]. However, lactobacilli and bifidobacteria are beneficial bacteria in both humans and animals since the final products of their sugar fermentation play a crucial role in establishing a positive network among bacterial groups of gut microbiota [69,70]. In the pig, this consideration is partially true since *Bifidobacterium* genus represents a small portion of the total bacteria of the gut microbiota, suggesting that other microbial groups play a crucial role in conferring benefits to the healthy animal [71,72]. Moreover, Sun et al. [73] described, in early-weaned piglets a reduction of *Lactobacillus* genus in the non-diarrheic compared to diarrheic animals. According to Sakdee et al. [16], our results shown no significant effects of tributyrin on the populations of *E. coli* and *Enterobacteriaceae*; on the contrary a significant reduction in *Lactobacillus* spp. and bifidobacteria has been observed. Similar decreases were obtained by sodium butyrate supplementation in jejunum samples collected in chicken. Indeed, using increasing doses of the supplementation, linear significant reduction in *Lactobacillus* counts was detected and the authors suggested an inhibitory effect of butyric acid on lactobacilli growth [74]. Tributyrin is neutral but after hydrolysis, one glycerol and three butyric acid molecules are released. As butyric acid is acidic, one of the possible hypotheses is that it acts by reducing the pH of the intestinal segment in which this hydrolysis occurs. Lactobacilli are resistant to low pH, but a sensitivity strain related could justified the reduction that we detected by real time PCR. Another possible explanation has been suggested by the results of an in vitro study conducted by Papon et al. [75]. In this paper, tributyrin showed a strong inhibitory effect on growth of *Lactobacillus* spp. strains. Interestingly, in that same study, no effects on cell growth of one *Lactobacillus curvatus* strain were detected in the presence of glycerol and butyric acid suggesting that tributyrin itself exert inhibitory action on bacterial cells. Moreover, Gresse et al. [76] demonstrated the dynamic of piglet microbiota across the intestinal tract, with the *Lactobacillus* genus having the highest relative abundance in the stomach, duodenum and ileum segments that are also the site in which tributyrin hydrolysis occurs [16]. We can suppose that the tributyrin exerts its inhibitory effects, before the hydrolysis, on sensitive *Lactobacillus* strains inhabiting these gastrointestinal segments. Concerning bifidobacteria, no equivalent studies focused on the tributyrin sensitivity of strains belonging to this genus are available in literature, but we can suppose similar inhibitory mechanisms or maybe a combination of low pH and butyric acid concentration. It is important to underline that the reduction, although significant, does not correspond to a dramatic drop in the content of both lactobacilli and bifidobacteria. Overall, for the *Lactobacillus* genus it seems that tributyrin has highlighted its gradual reduction that normally occurs in the weaning period [77] and for this reason, we take into consideration this modification as a signal that supplementation implies a gut microbiota modification.

However, considering both the positive effect of tributyrin supplementation on animal performance and the modification of microbial populations detected, we can suppose that tributyrin influenced some different relationships between beneficial bacteria. Indeed, in weaning piglets a negative correlation between *Lactobacillus* and Residual Feed Intake (RFI) value has been described by McCormack et al. [78] indicating that this genus is correlated with a higher feed efficiency. However, no significant differences in relative abundances between high and low feed-efficient pigs were detected, indicating that other groups of bacteria play a crucial role in energy diet exploitation. On the other hand, OTU related to *Butyrivibrio*, a butyrate-producing bacterium, with high ability to ferment complex carbohydrates, was identified only in stools of high feed-efficiency piglets. These data suggest that groups of bacteria other than lactobacilli and bifidobacteria play a more relevant role in feed efficiency because of their ability to digest cereal-based diets introduced during weaning.

These microbiological aspects require further investigations in order to better understand not only the ability of tributyrin to modulate the gut microbiota composition but also the bacterial interactions that can be correlated to the growth performance and animal health.

## 5. Conclusions

Nowadays, a considerable number of research projects are being conducted to develop, improve and implement nonpharmaceutical approaches for enhancing animal health and performance. Our study demonstrated that tributyrin increased the growth performance and feed efficiency without affecting the feed intake, indicating a major and effective utilization of nutrients. In particular, a comprehensive look at our findings showed that tributyrin increase the protein absorption, utilization and synthesis. In fact, we found an increase of serum albumin and insulin and a decrease in serum urea and faecal protein excretion, resulting in higher ADG and G:F. This is probably due to the multiple beneficial effects of tributyrin, especially the ability to lower the gastric pH, thus activating the pepsin enzyme, and the trophic effect on the gastrointestinal tract, resulting in a larger absorptive surface, which are both of great importance in the post-weaned piglets. The higher concentration of the HDL fraction, glucose and also insulin suggested that tributyrin could have also a regulatory effect on piglets’ metabolism. Despite the reduction of bifidobacteria and lactobacilli cell number, considered as beneficial groups of bacteria, animal performances and healthy status were not affected. We can conclude that tributyrin influences gut microbiota and may exhibit an inhibitory effect on some sensitive groups of bacteria. This supplementation could have beneficial effects on animal performance through the development of different relationships between groups of bacteria within the intestinal communities. All these data suggested that tributyrin could be considered a valuable feed additive for weaned piglets and, for these reasons, it will be interesting to evaluate and investigate in depth gut microbiota and bacterial interactions. 

## Figures and Tables

**Table 1:** Composition of the basal experimental diet. In the tributyrin (TRIB) group, 0.2% of corn was substituted with 0.2% tributyrin.

Items	Basal Diet
Ingredients	% as Fed Basis
Barley, meal	25.15
Wheat, meal	19.41
Corn, flakes	14.03
Corn, meal	4.85
Soybean, meal	4.65
Soy protein concentrates	4.11
Biscuits, meal	4.00
Dextrose monohydrate	3.50
Wheat middling	4.32
Whey protein concentrate	3.00
Fish, meal	2.50
Milk whey	2.50
Coconut oil	1.00
Soy oil	1.00
Plasma, meal	1.00
Organic Acids ^1^	1.00
Dicalcium phosphate	0.85
Animal fats	0.70
L-Lysine	0.50
Benzoic acid	0.40
L-Threonine	0.35
DL-Methionine	0.35
Sodium Chloride	0.27
Vitamins ^2^	0.25
L-Valine (96.5%)	0.15
L-Tryptophan	0.08
Flavouring ^3^	0.04
Copper sulphate	0.04
**Calculated Nutrient Levels ^4^**	
Crude protein	16.92
Ether extract	5.06
Crude fibre	3.15
Ash	5.1
DE ^5^ (Mc/Kg)	3.43

^1^ Organic acids: formic acid, sodium formate, sorbic acid, orthophosphoric acid, calcium formate, citric acid, and fumaric acid; ^2^ Vitamins and vitamin-like compounds per kg: vitamin A, 10,000; vitamin D3, 1000 IU; vitamin E, 100 mg; vitamin B1, 3 mg; vitamin B2, 96.3 mg; vitamin B6, 5.8 mg; calcium D-pantothenate, 27 mg; vitamin B12, 0.040 mg; vitamin K3, 4.8 mg; biotin, 0.19 mg; niacinamide, 35 mg; folic Acid, 1.4 mg. Choline chloride 120 mg, betaine chloride 70 mg; ^3^ vanilla flavouring; ^4^ calculation performed with Purimix software (Fabermatica, Cremona, Italy); ^5^ DE: digestible energy content estimated from NRC (2012).

**Table 2:** Weight gain and feed conversion rate of piglets fed on a diet supplemented with (TRIB; *n* = 60) or without (CTRL; *n* = 60) 0.2% tributyrin during the entire experimental period.

				*p*-Value			DF (Num DF; Den DF)		
Items ^1^	CTRL	TRIB	SE	TRT	DAY	TRT × DAY	TRT	DAY	TRT × DAY
**BW (kg)**				0.362	<0.001	<0.001	1; 10	3; 459	3; 459
day 0	8.70	8.76	1.04						
day 14	11.10	11.84	1.04						
day 28	15.40 ^a^	17.10 ^b^	1.04						
day 40	20.10 ^a^	23.20 ^b^	1.04						
**ADG (kg)**				0.034	<0.001	0.125	1; 10	2; 20	2; 20
day 0–day 14	0.171	0.220	0.03						
day 14–day 28	0.309	0.375	0.03						
day 28–day 40	0.395 ^a^	0.509 ^b^	0.03						
**ADFI (kg)**				0.095	<0.001	0.139	1; 10	4; 40	4; 40
day 0–day 14	0.327	0.426	0.03						
day 14–day 21	0.596	0.601	0.03						
day 21–day 28	0.797	0.901	0.03						
**G:F (%)**				0.049	0.189	0.160	1; 30	2; 30	2; 30
Overall	51.41 ^a^	56.53 ^b^	1.76						
day 0–day 14	52.44	50.75	3.06						
day 14–day 28	52.26	62.06	3.06						
day 28–day 40	49.54	56.79	3.06						

^1^ BW, body weight; ADG, average daily gain; ADFI, average daily feed intake; G:F, gain to feed ratio; TRT, treatment; DF, degree of freedom. Data are presented as least square means (LSMEANS) ± SE. Different superscripts indicate significant differences between groups (^a,b^: *p* ≤ 0.05).

**Table 3:** Serum concentrations of the different parameters analysed in piglets fed on a diet supplemented with (TRIB; *n* = 60) or without (CTRL; *n* = 60) 0.2% tributyrin.

Items ^1^	CTRL	TRIB	SE	*p*-Value	DF
Total protein content (g/L)	52.87	56.82	1.78	0.1314	21
Albumin (g/L)	19.30 ^a^	24.06 ^b^	0.76	0.0002	21
Globulin (g/L)	33.58	32.77	1.78	0.7504	21
A/G *	0.58 ^a^	0.78 ^b^	0.05	0.0117	21
Urea (mmol/L)	2.18 ^a^	1.08 ^b^	0.23	0.0026	21
ALT-GPT * (IU/L)	38.33	32.91	1.87	0.0528	21
AST-GOT * (IU/L)	54.16	48.27	4.10	0.3218	21
ALP * (UI/L)	165.67	194.00	16.68	0.2432	21
Total bilirubin (umol/l)	1.98	1.79	0.11	0.2425	21
Glucose (mmol/L)	5.00 ^a^	5.83 ^b^	0.27	0.0396	21
Total cholesterol (mmol/L)	2.50	2.73	0.09	0.1008	21
Calcium (mmol/L)	2.28	2.44	0.07	0.1068	21
Phosphorus (mmol/L)	3.05	3.19	0.10	0.3253	21
Magnesium (mmol/L)	0.84	0.87	0.02	0.3726	21
HDL (mmol/L)	0.76 ^a^	0.88 ^b^	0.04	0.0349	21
LDL (mmol/L)	1.60	1.73	0.07	0.1961	21
Creatinine (μmol/L)	78.91	72.18	4.79	0.3318	21
Triglycerides (mmol/L)	0.68	0.63	0.05	0.5331	21

* A/G, albumin/globulin; ALT-GPT, alanine aminotransferase; AST-GOT, aspartate aminotransferase; ALP, phosphatase alkaline; DF: degree of freedom. Data are presented as least square means (LSMEANS) ± SE. Different superscripts indicate significant differences between groups (^a,b^: *p* ≤ 0.05).
